# Proper Treatment of Common Eye Diseases

**Published:** 1881-12

**Authors:** 


### THE PROPER TREATMENT OF COMMON EYE DISEASES.

Dr. Samuel Theobald, in a report before the Medical and Chirurgical Faculty of Maryland, on the management of the commoner eye troubles, has the following to say about the use of astringents, boracic acid and yellow oxide of mercury:

The practice which at one time was so prevalent amongst physicians of prescribing for every sort of inflammation of the eye an astringent collyrium, is happily far less common than it formerly was, nevertheless, even at the present day it is not without its adherents. In reality, the field in which the use of astringents is called for is comparatively circumscribed, especially since it has been of late encroached upon on the one hand by the adoption of boracic acid as a remedy in ophthalmic therapeutics, and on the other hand by the more extended use which is being made of the yellow oxide of mercury. The presence of *excessive conjunctival secretion* is the true indication for the use of astringents, and the strongest contra-indication to their use is the existence of *decided ciliary irritation*. This is a rule of very general applicability, and one to which there are but few exceptions; if borne in mind, it may at least prevent some cases of iritis, or of corneal inflammation being aggravated by the injudicious use of nitrate of silver or sulphate of zinc collyria.

The astringents from which, according to my experience, the best results may be obtained, are sulphate of zinc, alum and nitrate of silver. The two former I have found better adapted to the milder, non-purulent varieties of conjunctival inflammation; the latter, to the purulent forms. Except in severe purulent conjunctivitis, my practice has been to employ very mild solutions. In catarrhal conjunctivitis I have seldom found it necessary to use a solution of sulphate of zinc stronger than one grain, or of alum stronger than two grains, to the ounce. Whenever indicated, as in ulceration of the cornea in purulent ophthalmia, instillations of atropia may be combined with the use of astringents, or the atropia may be added to the astringent solution, in the proportion of from one to four grains to the ounce.

Boracic acid and the yellow oxide of mercury, two valuable remedies in the treatment of diseases of the eye, to which reference has been made, though they do not properly belong under the head of astringents, may be spoken of in this connection. The use of the yellow oxide of mercury in the treatment of granular conjunctivitis, and pannus as a substitute for the astringents heretofore employed, has been strongly recommended in different quarters. Like all other remedies, it often fails to exert a controlling influence over this obstinate affection. I have, however, seen brilliant results from it in not a few cases. It is most conveniently applied rubbed up with vaseline, in the strength of one or two grains to the drachm, a small quantity being inserted between the upper or lower lid and the eye-ball once a day. To obtain permanent results the treatment must, of course, be kept up for a long time. Boracic acid, which was first brought prominently before the medical profession as a remedy in diseases of the eye, by my article upon the subject published in February of last year in the New York Medical Record, has since then come into more or less general favor in the treatment of those varieties of conjunctivitis in which astringents were previously used. The beneficial influence which it exerts in these cases is probably chiefly due to its antiseptic properties, although, as I have previously pointed out, its action is also slightly astringent, and it exhibits, moreover, a soothing or anodyne effect when applied to irritable eyes. In the treatment of purulent conjunctivitis it is especially valuable, as it renders unnecessary the use of the strong astringent solutions which were formerly employed; in catarrhal conjunctivitis also it is of great service. During the past year I have received from many quarters reports of favorable results obtained from this new agent by those who had been induced through my recommendation of it to make trial of it in the treatment of eye diseases. The accounts of its action in the purulent conjunctivitis of infants, have been particularly favorable. Although I have not been able to convince myself that advantage was gained in the treatment of this affection from using a solution stronger than four or five grains to the ounce, others, whose opportunities for testing the matter have been as good as my own, have thought that they obtained decidedly better results from saturated solutions (eighteen or twenty grains to the ounce). When the improvement from the use of the four or five grain solution of the acid has not been all that could be expected, I have added to it a very small quantity of sulphate of zinc (usually one grain to the ounce) with decided advantage. My opportunities for testing its action in granular ophthalmia have been very limited; it has not seemed to me, however, to exert a decided influence upon this intractable disease. In blenorrhœa of the lachrymal sac, and also in asthenopia accompanied by conjunctival hyperæmia, I constantly employ it with the happiest results.

